# Epithelial loss of mitochondrial oxidative phosphorylation leads to disturbed enamel and impaired dentin matrix formation in postnatal developed mouse incisor

**DOI:** 10.1038/s41598-020-77954-7

**Published:** 2020-12-16

**Authors:** Thomas Imhof, Katharina Rosenblatt, Galyna Pryymachuk, Daniela Weiland, Nicolas Noetzel, James Deschner, Olivier R. Baris, Sammy Kimoloi, Manuel Koch, Rudolf J. Wiesner, Yüksel Korkmaz

**Affiliations:** 1 Institute for Experimental Dental Research and Oral Musculoskeletal Biology, Center for Biochemistry, Medical Faculty, University of Cologne, Cologne, Germany; 2 Center for Physiology and Pathophysiology, Institute of Vegetative Physiology, Medical Faculty, University of Cologne, Robert-Koch-Strasse 39, 50931 Cologne, Germany; 3 Department of Anatomy, University of Cologne, Cologne, Germany; 4 Laboratory of Experimental Immunology, Institute of Virology, Faculty of Medicine and University Hospital Cologne, University of Cologne, 50931 Cologne, Germany; 5 Department of Periodontology and Operative Dentistry, University Medical Center of the Johannes Gutenberg University, Augustusplatz 2, 55131 Mainz, Germany; 6 Equipe MitoLab, UMR CNRS 6015, INSERM U1083, Institut MitoVasc, Université d’Angers, Angers, France; 7 Center for Molecular Medicine Cologne, Cologne (CMMC), University of Cologne, Cologne, Germany; 8 Cologne Excellence Cluster on Cellular Stress Responses in Aging-Associated Diseases (CECAD), Cologne, Germany

**Keywords:** Cell biology, Developmental biology, Physiology, Anatomy, Pathogenesis

## Abstract

The formation of dentin and enamel matrix depends on reciprocal interactions between epithelial-mesenchymal cells. To assess the role of mitochondrial function in amelogenesis and dentinogenesis, we studied postnatal incisor development in K320E-Twinkle^Epi^ mice. In these mice, a loss of mitochondrial DNA (mtDNA), followed by a severe defect in the oxidative phosphorylation system is induced specifically in Keratin 14 (K14+) expressing epithelial cells. Histochemical staining showed severe reduction of cytochrome c oxidase activity only in K14+ epithelial cells. In mutant incisors, H&E staining showed severe defects in the ameloblasts, in the epithelial cells of the stratum intermedium and the papillary cell layer, but also a disturbed odontoblast layer. The lack of amelogenin in the enamel matrix of K320E-Twinkle^Epi^ mice indicated that defective ameloblasts are not able to form extracellular enamel matrix proteins. In comparison to control incisors, von Kossa staining showed enamel biomineralization defects and dentin matrix impairment. In mutant incisor, TUNEL staining and ultrastructural analyses revealed differentiation defects, while in hair follicle cells apoptosis is prevalent. We concluded that mitochondrial oxidative phosphorylation in epithelial cells of the developed incisor is required for Ca2+ homeostasis to regulate the formation of enamel matrix and induce the differentiation of ectomesenchymal cells into odontoblasts.

## Introduction

The development of the dentin and enamel matrix depends on interactions between epithelial-mesenchymal and epithelial-epithelial cells^1,2^. The development of the mouse incisor begins with the formation of an epithelial thickening from the oral ectoderm at embryonic day E12. At E14, the epithelial thickening expands and invades the underlying neural crest-derived mesenchyme^3,4^. At E15, two epithelial cell structures termed cervical loops are formed in the incisor labial and lingual aspects. The epithelial cells in the cervical loop form a central epithelial stem cell containing core called stellate reticulum, that is surrounded on the inner side by the columnar epithelial cells of the inner enamel epithelium and on the outer side by the cuboidal epithelial cells of the outer enamel epithelium^5,6^. These epithelial stem cells enable continuous enamel formation throughout the lifetime of the animal^5,6^. The inner enamel epithelium cells differentiate into the preameloblasts which induce differentiation of the underlying neural crest-derived dental mesenchyme cells into odontoblasts^7^. The odontoblasts secrete the dentin matrix that in turn induces the differentiation of preameloblasts to ameloblasts to secrete the enamel matrix starting at E18^6,7^.

The differentiation of ameloblasts includes secretory, transition, and maturation stages^8,9^. The cuboidal epithelial cell layer underlying the secretory ameloblasts is termed stratum intermedium^8,10^. During the transition stage epithelial cells of the stratum intermedium, stellate reticulum, and outer enamel epithelium form the papillary cell layer^11–13^. Thus, in the secretory stage, the basal surface of ameloblast is attached to the cells of the stratum intermedium and in the maturation stage to the papillary cell layer^8,10^. The differentiation and functions of ameloblasts are regulated by cellular interactions between ameloblasts and stratum intermedium cells at the secretory stage and by cellular interactions between ameloblasts and the papillary cell layer at maturation stage^8,10,14,15^.

Mitochondria are under the control of two genomes, the nucleus encoding about 1200 proteins targeted to the organelles, and mtDNA, present in hundreds to thousands of copies in mammalian cells. Human mitochondrial DNA (mtDNA) is a 16.5 -kb circular double-stranded DNA molecule containing genes coding for 13 polypeptides essential for respiration and oxidative phosphorylation, 2 rRNAs, and a set of 22 tRNAs that are necessary for protein synthesis within the mitochondria^16^.

Depending on the differentiation stage, tooth epithelial cells alter their metabolism by adaptive changes of their mitochondrial equipment^17^. In cells of the inner enamel epithelium, stratum intermedium, and papillary cell layer developmental stage-dependent alterations in the localization of mitochondria were described. In the transition stage, the papillary cell layer is equipped with numerous mitochondria^11–13^. There are ultrastructural reports revealing a stage-related mitochondrial alteration in cytochrome c oxidase (COX) during the differentiation of ameloblasts^18^.

In addition to the transport of ions from the circulation to secretory ameloblasts through the stratum intermedium and to mature ameloblasts through the papillary layer, increased energy production by mitochondrial oxidative phosphorylation is also required for the removal of water and matrix proteins from the enamel matrix in mature ameloblasts^8,18^. However, it is not known how the enamel matrix formed by the interactions of the epithelial cell layers, when oxidative phosphorylation is disturbed only in these epithelial cells of the developed mouse incisor. In addition to tooth development^2,19–23^, epithelial-mesenchymal interactions regulate the development of all epidermal organs, including, whiskers, hair follicles and sebaceous glands^2,24^. We have shown previously a disturbed epithelial-mesenchymal cross-talk in developing hair follicles and sebaceous glands, when mitochondrial function was impaired in K14+ epithelial cells^25^. Thus, we asked ourselves, whether the disturbed mitochondrial function might also impair the interactions between epithelial and neural crest derived mesenchymal cells regulating the differentiation of ameloblasts and odontoblasts.

In order to study the role of mitochondrial function in enamel and dentin matrix formation of the mouse incisor, we impaired the mitochondrial respiratory chain by impairing mtDNA replication specifically in K14+ cells of all enamel epithelium cell layers. The K14 promoter was reported to initiate gene transcription in the whole ectoderm, including the oral epithelium starting from E10^26^. In the rapidly growing incisors, we found that mitochondrial function is required for the differentiation of secretory and mature ameloblasts, but also for the regulation of cellular interactions between secretory ameloblasts and epithelial cells of the stratum intermedium and between mature ameloblasts and epithelial cells of the papillary cell layer at the maturation stage. Proper epithelial mitochondrial function is also essential for interactions between inner enamel epithelium and neural crest derived mesenchymal cells for the differentiation of these cells into odontoblasts.

## Results

### COX/SHD-staining in cells of developing incisors

**Figure 1:**
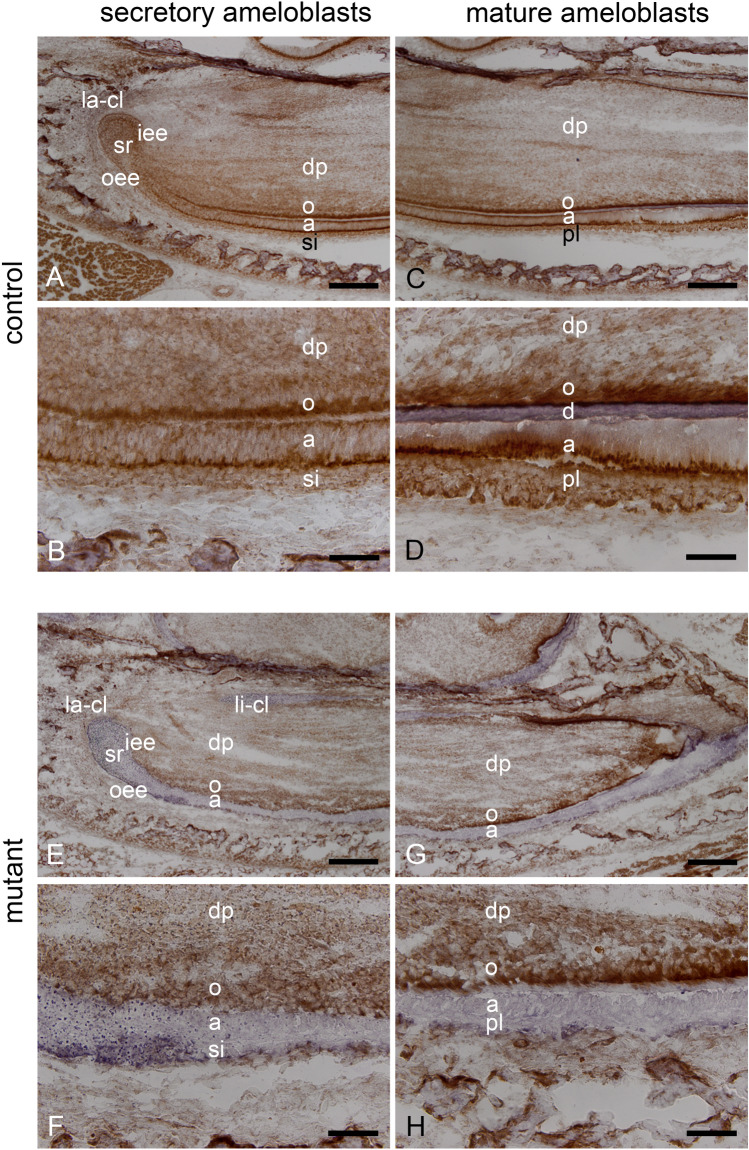
Histochemical localization of COX/SHD-staining in cells of the developing incisor of control and mutant K320E-Twinkle^Epi^ mice at P3. In P3 control mice COX/SHD is detected in stem cells of the cervical loop, in cells of the enamel epithelium and in odontoblasts (**A**–**D**). In P3 K320E-Twinkle^Epi^ mice COX/SHD staining is localized in cells of the dental papilla and in odontoblasts (**E**–**H**). However, no brown COX staining is found in the cervical loop or in the layers of the enamel epithelium (**E**–**H**). a, ameloblasts; dp, dental papilla; iee, inner enamel epithelium; la-cl, labial cervical loop; o, odontoblasts; oee, outer enamel epithelium; pl, papillary layer; si, stratum intermedium; sr, stellate reticulum; Scale bars: **A**,**C**,**E**,**G** = 200 µm; **B**,**D**,**F**,**H** = 50 µm.

In order to verify the specific epithelial loss of mitochondrial function in K320E-Twinkle^Epi^ mice we first analyzed the expression of the Cre and K14 transgene by immunohistochemistry (SFig. 1 & SFig. 2A–D) and also verified the loss of COX activity in skin sections (SFig. 2E–H) as previously described^27,28^. Next, COX/SHD activity was analyzed in P3 incisors and all epithelial and mesenchymal cells were positive for COX in control mice (Fig. 1A–D). In contrast, K320E-Twinkle^Epi^ incisors showed absence of COX activity, with essential subunits encoded by mtDNA, in the enamel epithelium (Fig. 1E–H). However, mesenchymal COX activity was intact, showing the tissue specificity of the Cre-mediated loss of mtDNA.

### Characterization of developing incisors by H&E staining

The secretory ameloblast layer of incisors of control mice at P0 (SFig. 3A) showed an elongated, columnar cell form lying on two up to three cuboidal stratum intermedium cell layers (SFig. 3B). The mature ameloblast cell layer from control incisors at P0 was identified in a proper cellular order lying on two up to three epithelial cell layers of the papillary cell layer (SFig. 3C). In contrast to the controls, the secretory ameloblasts of mutant K320E-Twinkle^Epi^ mice at P0 (SFig. 3D) were shorter and the stratum intermedium was thinner (SFig. 3E). The mature ameloblast cell layer of mutant K320E-Twinkle^Epi^ mice at P0 was disorganized and the papillary cell layer lost its continuity (SFig. 3F).

**Figure 2:**
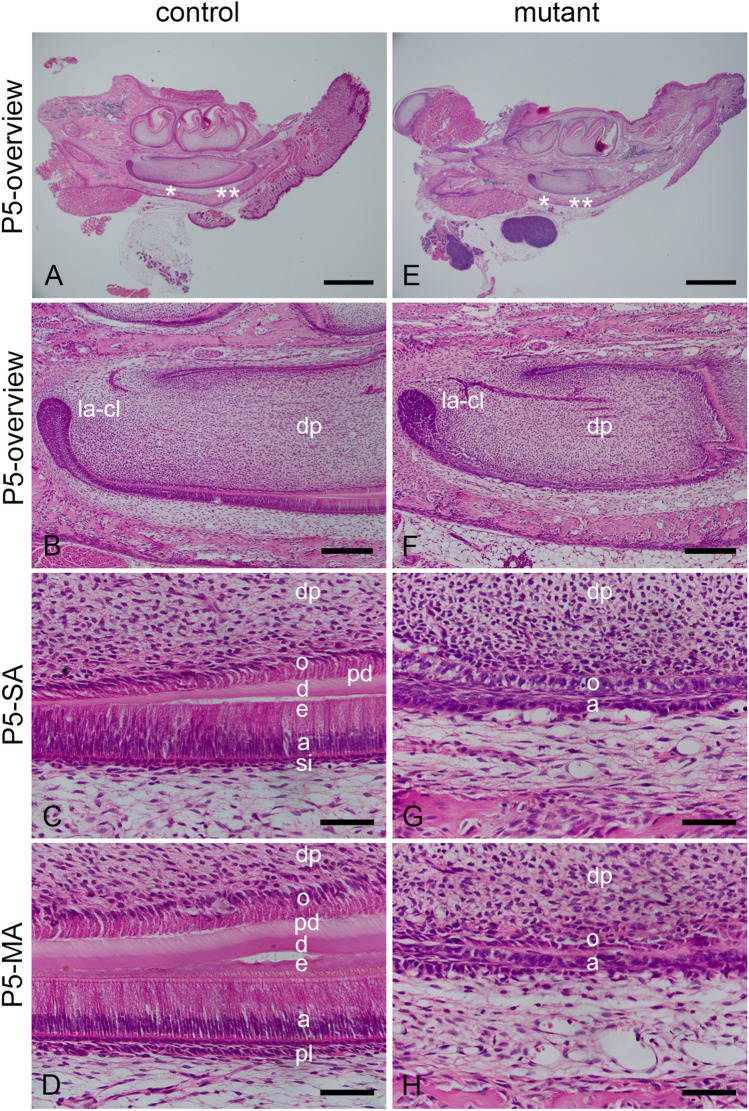
Hematoxylin and Eosin staining in cells of the developing teeth of control and mutant K320E-Twinkle^Epi^ mice at P5. The epithelial cells of the cervical loop (**A**,B), stratum intermedium (si) (**C**; the region is shown in A by one asterisk) and papillary cell layer (pl) (**D**; the region is shown in A by two asterisks) from control mice incisor at P5 are detected in a cellular order with two up to three cuboidal cell layers. In the P5 control, the odontoblasts are well ordered and structured (**C**,**D**). However, in the mutant mice at P5, the ameloblasts and odontoblasts are severely damaged (E–H). The labial cervical loop epithelium is still intact (**E**,**F**). In comparison with control mice at P5, the secretory ameloblasts (**G**; the region is shown in **E** by one asterisk) and mature ameloblasts (**H**; the region is shown in **E** by two asterisks) of mutant mice are shorter and disorganized. Compared to the control, the stratum intermedium (**G**) and papillary (**H**) layers are not identifiable of the mutant K320E-Twinkle^Epi^ mice at P5. In comparison with strong formation of enamel matrix at secretory (**C**) and especially at mature (**D**) stages in the control incisor, an enamel matrix is not detectable in incisor of the mutant K320E-Twinkle^Epi^ mice at P5 (**G**,**H**). a, ameloblasts; d, dentin; dp, dental papilla; e, enamel matrix; la-cl labial cervical loop; o, odontoblasts; pl, papillary layer; si, stratum intermedium; sr, stellate reticulum; Scale bars: **A**,**E** = 1 mm, **B**,**F** = 200 µm, **C**,**D**,**G**,**H** = 50 µm.

These phenotypic differences between control (Fig. 2A–D) and mutant (Fig. 2E–H) teeth were also investigated in P5 mice. In the control incisor, the cervical loop (Fig. 2A,B), the odontoblasts layer, dentin, enamel, secretory and mature ameloblasts, SI and PL were detected in a cellular and structural order (Fig. 2C,D & SFig. 4A-C). In K320E-Twinkle^Epi^ mice the cervical loop is well developed (Fig. 2E,F). However, the committed epithelial cell layers are strongly affected (Fig. 2G,H & SFig. 4D-F). The pre-odontoblasts have also lost their stratified organization and polarity (Fig. 2F,G) and close to cervical loop and no enamel or dentin formation is observed. Further analysis younger animals showed that, in comparison to the control incisor (SFig. 5A, B), the development of odontoblast and ameloblast cells was already strongly affected in P3 old mice (SFig. 5C, D). The most striking finding in the P3 old mice was that the dentin formation stopped in the newly developed apical incisor region (SFig. 5C, D).

### von Kossa staining of the extracellular dentin and enamel matrix of developing incisors

**Figure 3:**
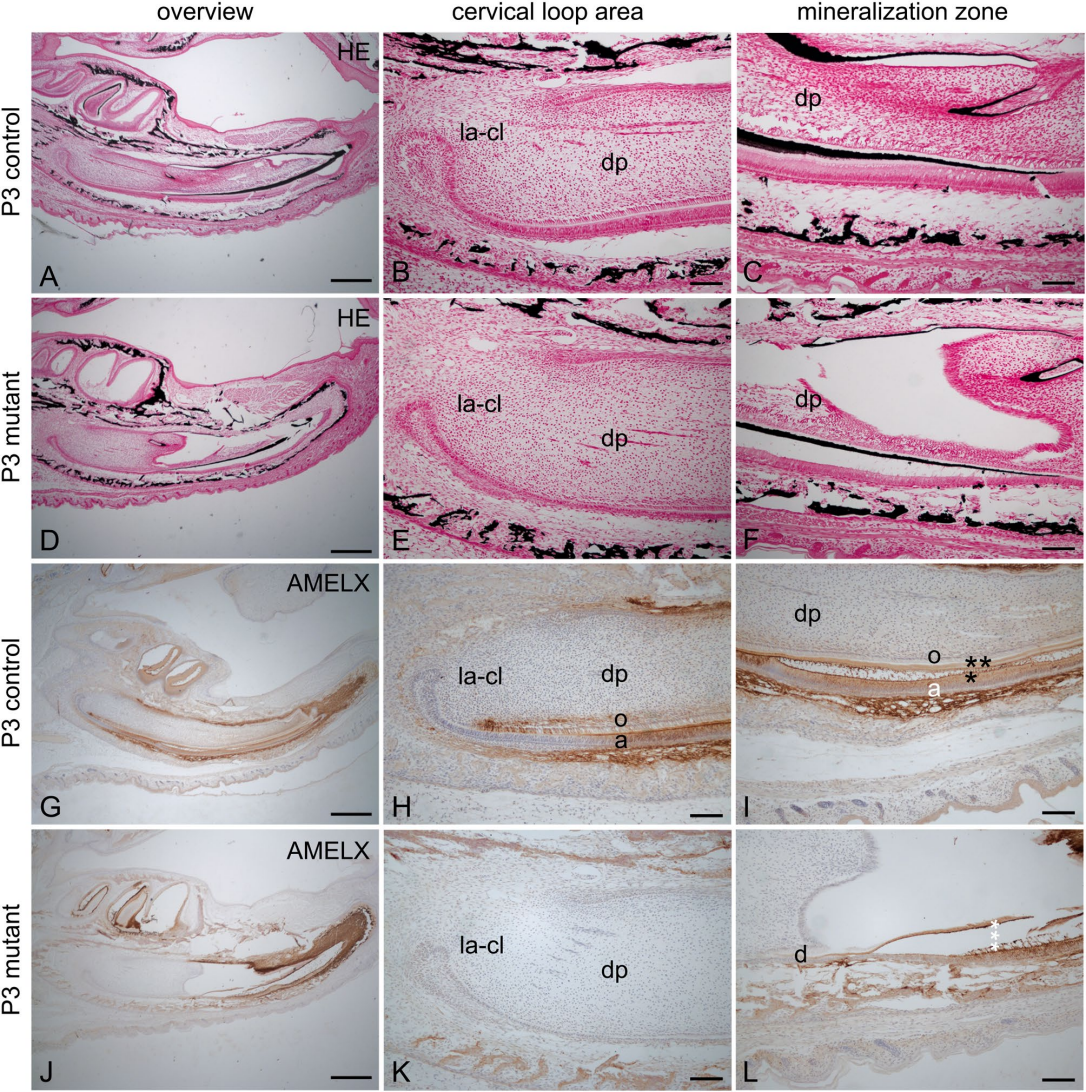
Von Kossa staining and localization of amelogenin and in the developing incisors of control and mutant P3 K320E-Twinkle^Epi^ mice. In P3 control incisors (**A**–**C**) the thick extracellular dentin and enamel matrices are positive for von Kossa staining (black) overlying the secretory stage ameloblasts (**C**). In comparison to the control, von Kossa staining in K320E-Twinkle^Epi^ mice (**D**–**F**) showed only a thin dentin matrix layer at the secretory stage ameloblast region (**F**) in developing incisor of mutant mice at P3. Amelogenin is detected with a strong staining intensity in P3 control ameloblasts and enamel matrix (**G**–**I**). In the apical incisor, amelogenin is additionally expressed by pre-odontoblasts (**G**,**I**). Furthermore, amelogenin is localized in the incisor dentin matrix (**I**, two asterisks) and in enamel matrix (**I**, one asterisk). In P3 K320E-Twinkle^Epi^ mice incisors (**J**–**L**) no amelogenin expression is found in the apical secretory stage ameloblasts nor in the pre-odontoblasts (**K**). In comparison to the control, amelogenin is identified only and with weakly staining intensities in ameloblasts of the secretory stage ameloblasts (**L**). The three asterisks show the cracked dentin layer, which has a strong unspecific coloration due to section detachment (section margin effect) (**L**). la-cl, labial cervical loop; a, ameloblasts; d, dentin; dp, dental papilla; e, enamel matrix; o, odontoblasts. Scale bars: **A**,**D**,**G**,**J** = 500 µm, **B**,**C**,**E**,**F**,**H**,**I**,**K**,**L** = 100 µm.

In developing incisors of control mice at P3, strong von Kossa staining was detected in the mineralized extracellular dentin and enamel matrix (Fig. 3A–C). In comparison to the control, mutant mice showed a very thin staining which was identified mainly in the extracellular dentin matrix (Fig. 3D–F).

### Expression of amelogenin in cells of developing incisors

The organic matrix of enamel contains 90% amelogenin, 8–10% ameloblastin and trace amounts of enamelin^8^. In P3 control mice amelogenin expression was detected in pre-odontoblasts and in the epithelial layer starting with the differentiation to ameloblasts (Fig. 3G–I). In contrast to that, the P3 K320E-Twinkle^Epi^ showed a complete loss of amelogenin expression in the newly formed mesenchymal and epithelial cells. In the more incisal region of the epithelial layer the ameloblasts were positive for amelogenin but in contrast to the control, no enamel layer formation was found (Fig. 3J–L).

### TUNEL staining and ultrastructure of developing incisors

**Figure 4:**
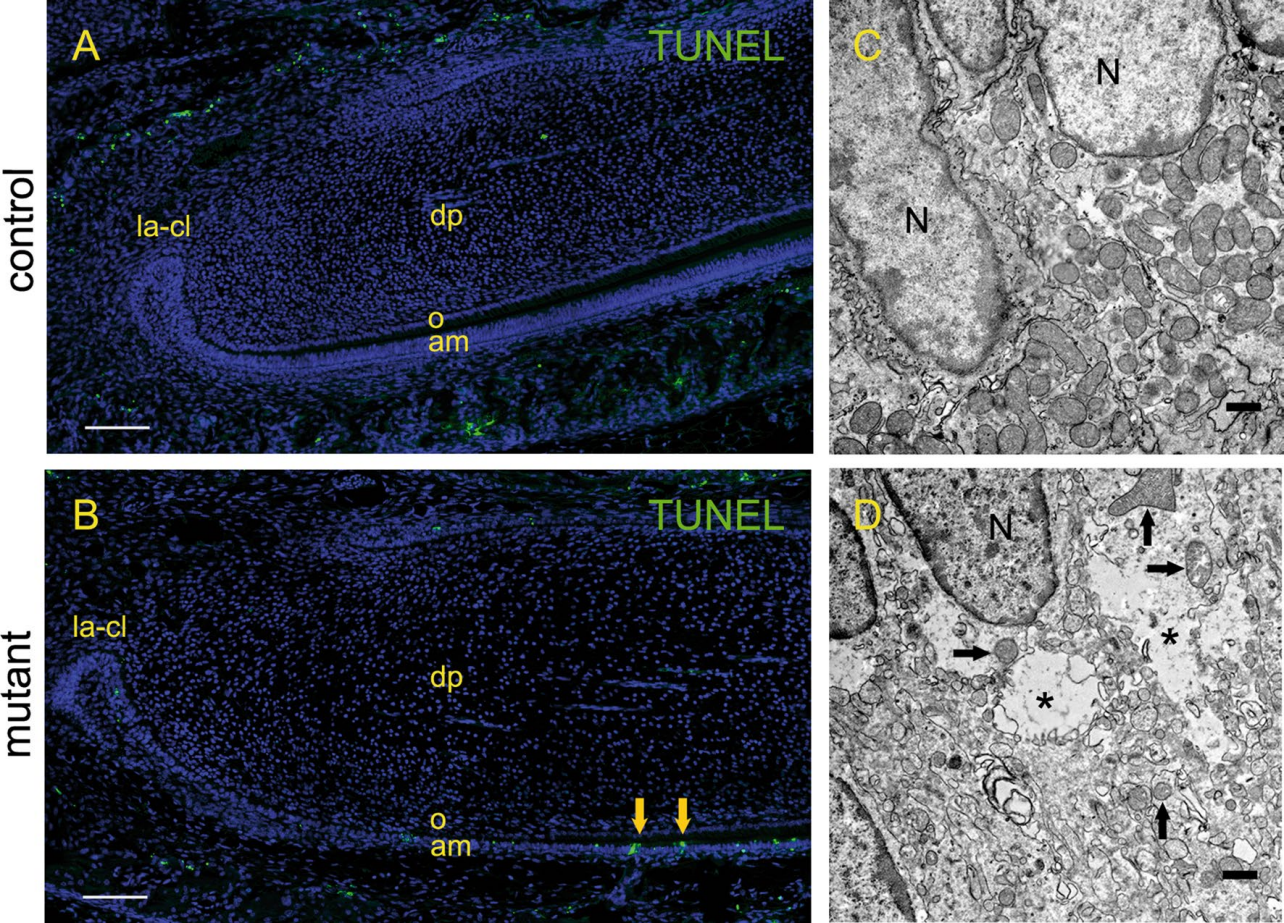
TUNEL staining and ultrastructure of the developing incisor from control and mutant K320E-Twinkle^Epi^ mice. In the enamel epithelium of P3 control incisors, ameloblasts and the stratum intermedium cells are well distinguishable and no specific TUNEL staining is visible. In K320E-Twinkle^Epi^ however, the epithelial layer organization is less clear and few TUNEL positive (green) are found in ameloblasts and stratum intermedium cells. In ultra-thin section from the secretory ameloblast region of the control mouse incisorat P5, numerous mitochondria with cristae, membrane structures and nuclei are detected in one order (**B**). In the secretory ameloblast region of the mutant incisor, the mitochondria are strongly disturbed (arrow), the chromatin is altered and the membrane structures are disorganized in a necrotic manner (**D**). Areas of decay (asterisks) in the cytoplasm and numerous damaged organelles indicate necrotic changes (**D**). a, ameloblasts; dp, dental papilla; iee, inner enamel epithelium; la-cl, labial cervical loop; o, odontoblasts; TUNEL, terminal deoxynucleotidyl transferase dUTP nick end labeling. Scale bars: **A**,**C** = 100 µm; **B**,**D** = 500 nm.

In P3 control incisors no specific TUNEL staining was detected in the enamel epithelium nor in the mesenchymal tissue (Fig. 4A). In contrast, in K320E-Twinkle^Epi^ mice few nuclear staining, which is specific for apoptosis, was found (Fig. 4B).

In ultra-thin sections, ameloblasts and their membrane structures were detected in an orderly manner (Fig. 4C). In control mice, at the transitional region between ameloblasts and stratum intermedium cells, numerous mitochondria were densely packed and observable in the cytoplasm around the nucleus in comparable sizes and with regular cristae structures (Fig. 4C). In mutant mice, the membrane structures of the organelles were disorganized and only a few damaged mitochondria of different sizes and without cristae structures were visible in the cytoplasm (Fig. 4D).

## Discussion

The number of mitochondria a cell contains depends on the function of the different cell types with different energy requirements^18,29^. We have shown recently that, quite surprisingly, severe mitochondrial dysfunction in the highly proliferative basal cell layer of the epidermis does not result in a proliferation defect^27,28^, while differentiation of skin appendages like hair follicles and sebaceous glands was dramatically impaired^25^. In this study we have studied the consequences of a severe impairment of the mitochondrial respiratory chain in epithelial cells of the incisor by expressing a dominant-negative mutant of the mitochondrial replicative helicase Twinkle, causing the complete loss of mtDNA in rapidly proliferating cells^27,28^. We found that this not only affects the primarily targeted epithelial cells and epithelial cell–cell interactions at secretory (between ameloblasts and the stratum intermedium) and maturation stages (between ameloblasts and the papillary cell layer), but obviously also affects cell–cell interactions between the epithelium and neural crest-derived mesenchymal cells. Although the respiratory chain was impaired in all epithelial cells of the developing incisor, the most severe developmental defects were observed in the epithelial cells of the stratum intermedium, the papillary cell layer and surprisingly in the odontoblast differentiation. The defects we have found in the epithelium became more pronounced at P5 compared to P0. This indicated that the metabolic activities of the differentiated enamel epithelium cells, in comparison to epithelial progenitor cells of the developing incisor, are strongly dependent on mitochondrial function.

The skin, a previously studied ectodermal organ, showed that the loss of mtDNA is associated with increased apoptosis^28^ as described by TUNEL staining. TUNEL staining in the nucleus is generally associated with apoptosis, while staining in the cytoplasm reveals programmed necrosis^30,31^. TUNEL staining in K320E-Twinkle^Epi^ incisors shows few apoptotic epithelial cell apoptosis in the ameloblast region (Fig. 4A,C). However, no apoptotic cells were found in the pre-ameloblast region. We further verified the loss of mitochondria in ultrathin sections of P5 incisors. K320E-Twinkle^Epi^ incisor ameloblasts showed that the mitochondria and the membrane structures of the cell organelles and cells were severely disturbed. An intact plasma membrane is considered a hallmark for apoptotic cell death, while broken and damaged membranes indicate programmed necrosis^32^. This indicates that the absence mtDNA in older K320E-Twinkle^Epi^ incisors might lead to death of differentiated cells. Based on this defect, we assume that the loss of mtDNA in the incisor primary leads to a differentiation defect of all pre-differentiated enamel epithelial cells. Our findings in newborn mice (SFigure 3) support that hypothesis; here secretory stage ameloblasts are still found in the apical region of the incisor. However, those cells are shorter and the underlying stratum intermedium is less pronounced. In P3 old mice this part of the incisor has erupted to more incisal regions of the tooth. Three days old K320E-Twinkle^Epi^ mice express amelogenin in this region, but no mineralized enamel is found in von Kossa stainings (Fig. 3). In the more apical region of the P3 K320E-Twinkle^Epi^ incisor were the cells were just recently developed, the ameloblasts failed to express amelogenin and the stratification of the epithelium is lost.

Intact function of the stratum intermedium is required for survival and differentiation of secretory ameloblasts^33^. For example, the stratum intermedium cells produce alkaline phosphatase regulating differentiation of secretory ameloblasts^34,35^. Also, mitochondrial function in epithelial cells of the stratum intermedium was implicated to be essential for active ion transport between secretory ameloblasts and stratum intermedium cells^13,36^. In mice lacking homeobox-containing transcription factor Msx-2, stratum intermedium cells are not well formed and ameloblast function is impaired^37^. The secretory function of ameloblasts is regulated by sonic hedgehog which is produced by cells of the stratum intermedium^7^. In addition, the formation of cellular junctions between inner enamel epithelium and stratum intermedium cells is regulated by nectin^14^. Since loss of the respiratory chain in epithelial cells induces differentiation defects in both secretory ameloblasts and epithelial cells of the stratum intermedium, these cells can no longer function alone or interact with their neighbors. In order to transport ions (Ca^2+^, PO_4_^3-^) from the circulation to secretory ameloblasts via the stratum intermedium^8,38^, a high amount of energy is required provided by mitochondrial oxidative phosphorylation^8,18^. Due to severe defects in developed epithelial cells of the stratum intermedium, when mitochondrial function in K14+ epithelial cells is disturbed, we assumed that disturbed mitochondrial function leads to loss of mitochondrial oxidative phosphorylation in the epithelial cells of the stratum intermedium and in secretory ameloblasts. Since the required energy is not available in the epithelial cells of the stratum intermedium and in the secretory ameloblasts, there is no active ion transport to the developing secretory ameloblasts.

During biomineralization of the enamel matrix, amelogenin regulates the orientation, shape and length of enamel hydroxyapatite crystals^39,40^. In mice in which the amelogenin gene was deleted, only a thin mineralized matrix without prismatic structure was detected^41^. In comparison to these findings, we observed not only a strongly reduced formation of amelogenin, but also a strong disturbance in the formation of the enamel matrix in mutant mice. This suggests that the formation of amelogenin does not take place due to the impaired oxidative phosphorylation and that amelogenin may play an important role in the biomineralization of the enamel matrix. The expression of amelogenin has been reported to be Ca^2+^ dependent^42,43^. The lack of von Kossa staining in the enamel matrix of mutant mice (Fig. 3M–P) indicates a disturbed Ca^2+^ deposition in the enamel matrix. Therefore, we assume that Ca^2+^ metabolism in the enamel matrix might be defective due to impaired mitochondrial function and that ameloblasts are not able to produce amelogenin.

Membrane ion channels are involved in the transport of ions into and out of the cells regulating cell–cell and cell–matrix interactions between mature ameloblasts and the papillary cell layer^13,36^. In addition to active ion (Ca^2+^ and HCO^3−^) transport from the circulation to the mature ameloblasts via the papillary layer, the removal of water and matrix proteins from the enamel matrix requires high ATP production by mitochondrial oxidative phosphorylation in mature ameloblasts^8,18^. Since the required ATP is not available due to damaged oxidative phosphorylation in epithelial cells of the papillary layer and in mature ameloblasts, we assume that the active transport of Ca^2+^ ions from the circulation via the papillary layer to the mature ameloblasts but also the removal of the water and the proteins from the enamel matrix is impaired. In intact cells, mitochondria and endoplasmic reticulum store Ca^2+^ and release Ca^2+^ into the cytosol as needed^8,44^. An important contributor to Ca^2+^ entry is the store-operated Ca^2+^ entry pathway via the Ca^2+^ release activated Ca^2+^ channels mediated by STIM1 and ORAI proteins^29,44^. The broken and disturbed membrane structures of mutant ameloblasts and cells of the papillary cell layer due to programed cell death may be the result in defects of Ca^2+^ channels so that Ca^2+^ homeostasis and therefore biomineralization of the enamel matrix might be strongly disturbed. It is possible that this entire Ca^2+^ metabolism does not take place in the mutated enamel matrix. The mutations in STIM1 and ORAI1 genes inducing multiple defects in the biomineralization of the enamel matrix^29^ support our assumption. The defects in biomineralization of the enamel matrix of mutant K320E-Twinkle^Epi^ mouse incisor highlight the requirement of mitochondrial function in mature ameloblasts and in epithelial cells of the papillary cell layer in cell–cell and cell–matrix interactions during the maturation stage.

In tooth development, amelogenins make up 90% of the organic matrix of the tooth enamel protein and the different splice variants of amelogenin produced by preameloblasts and odontoblasts may regulate the interactions between preameloblasts and ectomesenchymal cells of the dental papilla^45,46^. It has been described that small splice variants of amelogenin have signaling activity controlling mesenchymal differentiation and bone formation in vivo and the ectomesenchymal cells of the dental papilla need ameloblast-derived amelogenin in order to differentiate into odontoblasts^46,47^. In comparison to the control (Fig. 3G–I), in the apical secretory stage ameloblasts and in pre-odontoblasts of the K320E-Twinkle^Epi^ mice incisor no amelogenin was detected (Fig. 3J–L). In ameloblasts of the secretory stage of mutant incisor amelogenin was only weakly identified (Fig. 3J–L). The reduced amelogenin expression might be due to disturbed Ca^2+^ homeostasis and signaling or defective protein synthesis. The neural crest derived mesenchymal cells of the dental papilla cannot receive an induction of epithelial-derived signaling cues such as small amelogenin splice variants^46,47^ and/or FGF9^2,20,21,48^. In mutant P3 incisors, we found odontoblast differentiation defects and no apical predentin matrix deposition. Based on the K14Cre-mediated epithelial mtDNA loss, our hypothesis is that altered epithelial-mesenchymal crosstalk might be the reason for the odontoblast phenotype.

In the context of cell–cell interactions during organ development, our results are important not only for the interactions between epithelial-epithelial, but also for the interactions between epithelial-mesenchymal cells. Due to severe defects in epithelial cells of the developed incisor and lacking of cellular and extracellular amelogenin, when mitochondrial function in K14 + epithelial cells of the developed mouse incisor is disturbed, we concluded that mitochondrial function is essential for Ca^2+^ homeostasis regulating the synthesis of amelogenin, to form enamel matrix and to induce differentiation of ectomesenchymal cells into odontoblasts. Whether a cellular selective impaired function only in neural crest derived mesenchymal cells can lead to an impairment of the enamel matrix formation should be clarified with additional experiments in cell specific conditional knockout mouse model using cre/loxP method.

## Methods

### Statement on animal use and care

The animal protocols were approved by the animal care committee of the University of Cologne and local government authorities (Bezirksregierung Koeln; Landesamt fuer Natur, Umwelt und Verbraucherschutz (LANUV), Recklinghausen, Az: 84-02.04.2013.A190) and all methods were performed in accordance with the ethical guidelines and regulations.

### Generation and genotyping of K320E-*Twinkle*^*Epi*^ mice

K320E-Twinkle^Epi^ mice were generated by crossing K14-Cre mice^49^ with R26-TwinkleK320E^loxP/+^ mice^28^. Cre-recombination leads to the expression of both K320E-Twinkle and green fluorescent protein (GFP) in the epithelial cells. Littermates with the genotype R26-K320E-Twinkle^loxP/+^, which do not harbor a K14-cre, were used as controls. Genotyping was performed using genomic DNA from tail biopsies. The specificity of Cre-recombination was checked by PCR using total tissue DNA extracted with the DNeasy Blood & Tissue Kit (Qiagen, Hilden, Germany)^28^. The expression of Cre-recombinase in K14+ epithelial cells of mouse skin sections was tested by immunohistochemistry using an antibody against Cre-recombinase.

### Tissue preparation

Control and mutant mice (n = 3) were sacrificed at P0, P3, and P5 (owing to their postnatal lethality at or shortly after 5 d) by decapitation. For COX/SDH staining, unfixed P3 heads (n = 3) were embedded in OCT Compound (Sakura, Staufen, Germany) and cryosectioned at 10 µm. For immunohistochemical staining, heads (n = 3) were immersion-fixed in 0.1 M phosphate-buffered saline (PBS), pH 7.4 containing 4% paraformaldehyde (PFA) for 48 h. Then, half of the lower and upper jaws were dissected and demineralized for 5 d in 10% EDTA at 4 °C. The samples were then washed with 0.1 M PBS, pH 7.4 and embedded in paraffin.

### Hematoxylin and Eosin (H&E) staining

Paraffin Sects. (5 µm) were deparaffinized in xylene, rehydrated in descending ethanol series, rinsed in distilled water, and stained by H&E staining.

### von Kossa staining

To detect calcium phosphate crystals in the enamel matrix, the non-decalcified sections were stained with the von Kossa method. Paraffin Sects. (5 µm) of mouse jaws were treated with 3% silver nitrate and exposed to ultraviolet light for 30 min. Then, the sections were washed with distilled water. Thereafter sections were treated with 5% sodium thiosulfate for 3 min, washed with distilled water and counterstained with Nuclear Fast Red solution.

### Cytochrome c oxidase (COX)/Succinate dehydrogenase (SDH) double-labeling histochemistry

Cryosections of mouse jaws were sequentially stained for cytochrome c oxidase (COX, complex IV; containing 3 mtDNA subunits; brown stain) as well as succinate dehydrogenase (SDH, complex II; containing only nuclear encoded subunits; blue stain) activity as previously described^53^. In short, the sections (10 µm) were dried for 1 h and treated with 250 µl COX-histochemical solution in 0.1 M PBS (pH 7.0) containing 1 × 3,3’-diaminobenzidine, 100 µM cytochrome c and 2 µg/ml bovine catalase for 40 min at 37 °C. Sections were washed 4 × 10 min in 0.1 M PBS. Subsequently, sections were treated with SDH-histochemical solution containing 1.5 mM NBT, 130 mM sodium succinate, 0.2 mM PMS, and 1.0 mM sodium azide in 0.1 M PBS (pH 7.0).

### Terminal deoxynucleotide transferase-mediated deoxyuridine nick end labeling (TUNEL) assay

Paraffin sections (5 µm) were stained according to the Promega DeadEnd™ Fluorometric TUNEL System manufacturer protocol. Pictures were acquired with a Leica SP8 confocal microscope.

### Immunohistochemistry

The deparaffinized sections were washed in 0.05 M Tris buffered saline (TBS) and the endogenous peroxidase activity inhibited by 0.05 M TBS containing 3% H_2_O_2_. Unspecific binding sites were blocked with 10% normal goat serum (Vector Laboratories, Burlingame, CA). The sections were then incubated with the following primary antibodies: rabbit anti-K14 (1:500; Berkeley Antibody Company, Richmond, CA), biotinylated mouse anti-amelogenin (1:500; Santa Cruz Biotechnology), and rabbit anti-Cre recombinase protein (1:800; Cell Signaling Technology, Frankfurt am Main, Germany). The specific signal was detected by the use of biotinylated secondary antibodies and HRP conjugated streptavidin conjugates (Vector Laboratories). A negative control was performed by incubations without primary antibodies.

### Transmission electron microscopy

The dissected maxillae and mandibles of mice at P5 were immersed in a fixative containing 3% glutaraldehyde and 0.1 M cacodylate buffer (pH 7.2) at 4 °C for 28 h and decalcified in 10% ethylenediaminetetraacetic acid (EDTA) at 4 °C for 5 d. The samples were washed in 0.1 M sodium cacodylate buffer for 48 h at 4 °C, post-fixed with 1% OsO_4_ (osmium tetroxide), dehydrated through acetone and embedded in araldite CY212 (Durcupan ACM, Fluka). The semithin sections were cut at a thickness of 1 µm and stained with methylene blue. The ultrathin sections were cut at 70 nm using an ultra-microtome (Reichert-Nissei, Unterschleißheim, Germany) with a diamond knife, mounted on copper grids, and contrasted 10 min with saturated 2% uranyl acetate in 70% ethanol followed by 5 min with 0.2% aqueous lead citrate, pH 11.8. The stained grids were examined using a FEI Tecnai G20 TEM (Hillsboro, OR).

## Supplementary information

Supplementary Figures.
