# Supplementary material for: Epithelial loss of mitochondrial oxidative phosphorylation leads to disturbed enamel and impaired dentin matrix formation in postnatal developed mouse incisor
Source: Sci Rep. 2020 Dec 16;10:22037. doi: 10.1038/s41598-020-77954-7 (PMC7744519; doi:10.1038/s41598-020-77954-7)

**Supplement:**

**Epithelial loss of mitochondrial oxidative phosphorylation leads to disturbed enamel and impaired dentin matrix formation in postnatal developed mouse incisor**

Thomas Imhof^1^, Katharina Rosenblatt^2^, Galyna Pryymachuk^3^, Daniela Weiland^2^, Nicolas Noetzel^2^, James Deschner^4^, Olivier R. Baris^5^, Sammy Kimoloi^2^, Manuel Koch^1,6^, Rudolf J. Wiesner^2,6,7*^, Yüksel Korkmaz^4*^

^1^Institute for Experimental Dental Research and Oral Musculoskeletal Biology, Center for Biochemistry, Medical Faculty, University of Cologne, Cologne, Germany

^2^Center for Physiology and Pathophysiology, Institute of Vegetative Physiology, Medical Faculty, University of Cologne, Cologne, Germany

^3^Department I of Anatomy, University of Cologne, Cologne, Germany

^4^Department of Periodontology and Operative Dentistry, University Medical Center of the Johannes Gutenberg University, Mainz, Germany

^5^Equipe MitoLab, UMR CNRS 6015, INSERM U1083, Institut MitoVasc, Université d'Angers, Angers, France

^6^Center for Molecular Medicine Cologne, Cologne (CMMC), University of Cologne, Cologne, Germany

^7^Cologne Excellence Cluster on Cellular Stress Responses in Aging-Associated Diseases (CECAD), Cologne, Germany

***corresponding authors:**

Rudolf J. Wiesner

Center for Physiology and Pathophysiology,

Institute of Vegetative Physiology, Medical Faculty, University of Cologne, Cologne

Robert-Koch-Strasse 39, 50931 Cologne, Germany

E-mail: rudolf.wiesner@uni-koeln.de

Yüksel Korkmaz

Department of Periodontology and Operative Dentistry,

University Medical Center of the Johannes Gutenberg University

Augustusplatz 2, 55131 Mainz, Germany

E-mail: [yueksel.korkmaz@unimedizin-mainz.de](mailto:yueksel.korkmaz@unimedizin-mainz.de)

**Supplementary Figure Legends:**

**SFigure 1.** Localization of Cre-recombinase in the basal epithelial cells of the K14 Cre^+^ mice at P0.

The immunohistochemical incubation of the consecutive skin sections (A, B) of K14Cre^+^ mice at P0 with antibody to the Cre-recombinase revealed its localization in epithelial cells at the basal layer and hair follicle indicating cell specific activity of Cre-recombinase in epithelial cells of K14 Cre^+^ mice (C). In the control incubations without Cre-recombinase antibody, immunohistochemical localization of Cre-recombinase is absent in cells of the K14Cre^+^ mice (D). Scale bars: A, B= 1 mm; C, D= 50 µm.

**SFigure 2.** K14-immunoreactivity and COX/SHD-staining in head epidermal cells of control and mutant K320E-Twinkle^Epi^ mice at P0.

The epidermal basal cell layer and hair follicle cells revealed localization for an immunoreactivity of K14 of the control (A, B) and mutant (C, D) mice at P0. In the epidermal basal cell layer and hair follicles cells of control mice, a staining for COX/SHD is detected (E, F). A histochemical staining for COX/SHD was absent in the epidermal basal cell layer and in hair follicle cells of mutant mice at P0 (G, H).

bl, basal layer; e, epidermis; hf, hair follicle; lp, lamina propria. Scale bar: A-H= 50 µm.

**SFigure 3.** Hematoxylin and Eosin staining in cells of the developing teeth of control and mutant K320E-Twinkle^Epi^ mice at P0.

(A-C) The stratum intermedium (si) (B; the region is shown in A by one asterisk) and papillary cell layer (pl) (C; the region is shown in A by two asterisks) from control mice incisor at P0 are detected in a cellular order with two up to three cuboidal cell layers. (D-F) In comparison with control mice at P0, the secretory ameloblasts (E; the region is shown in D by one asterisk) and mature ameloblasts (F; the region is shown in D by two asterisks) of mutant mice are shorter and disorganized. The si (asterisk; E) and pl (asterisk; F) are thinly identified of the mutant K320E-Twinkle^Epi^ mice at P0. Compared to the strong formation of enamel matrix at maturation stage in the control mice (C), an extremely thin enamel matrix is detected in incisor of the mutant K320E-Twinkle^Epi^ mice (E) at P0. In comparison to the control (C) dentin matrix of mutant animals (F) is thinly formed.

a, ameloblasts; d, dentin; dp, dental papilla; e, enamel matrix; la-cl labial cervical loop; o, odontoblasts; pl, papillary layer; si, stratum intermedium; Scale bars: A, D= 1 mm, B, C, E, F= 50 µm

**SFigure 4.** Semi thin sections of control and mutant incisors of mice at P5.

In the control overview (A) and detailed (B, C) images the odontoblasten layer, dentin, enamel, secretory and mature ameloblasts, stratum intermedium and papillary layer are well structured. In the mutant overview (D) and detail (E, F) images it can be clearly seen that the width of the layers for odontoblasts, dentin, enamel, secretory and mature ameloblasts, stratum intermedium and papillary layer of mutant incisor is significantly thinner (D-F) compared to the width of the layers of the control (A-C). The formation of the enamel is strongly disturbed (F).

d, dentin; dp, dental papilla; e, enamel; ma, mature ameloblasts; o, odontoblasts; pd, predentin; pl, papillary layer; sa, secretory ameloblasts; si, stratum intermedium. Scale bars: A, D= 1 mm; B, C, E, F= 50 µm.

**SFigure 5.** Hematoxylin and Eosin staining in cells of the developing teeth of control and mutant K320E-Twinkle^Epi^ mice at P3.

In the control overview (A) and detailed (B) images the odontoblast and ameloblast layer are well structured. In the mutant overview (C) and detail view (D) it can be clearly seen that the odontoblasts (asterix) are differentiating and that no dentin formation is visible.

a, ameloblasts; dp, dental papilla; la-cl, labial cervical loop; o, odontoblasts. Scale bars: A, C= 100 µm; B, D= 50 µm.

**SFigures:**

SFigure 1


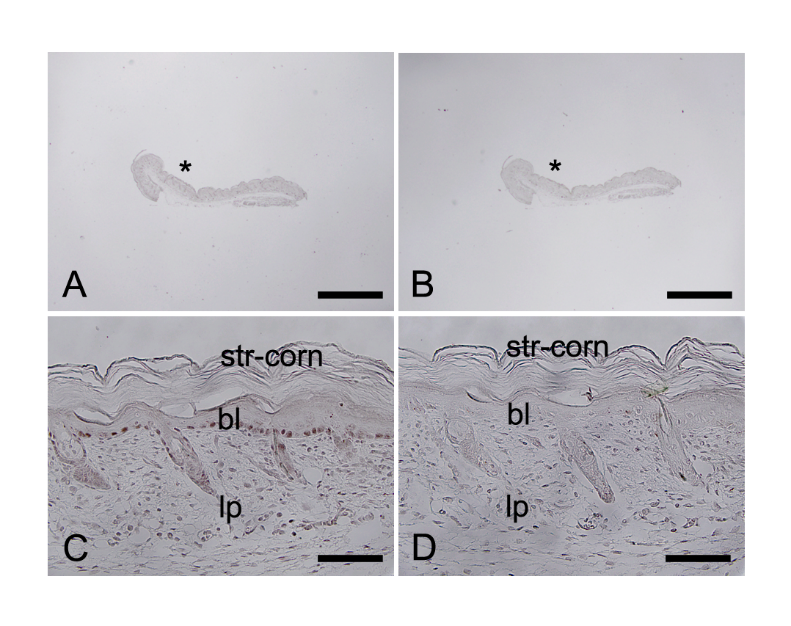


SFigure 2


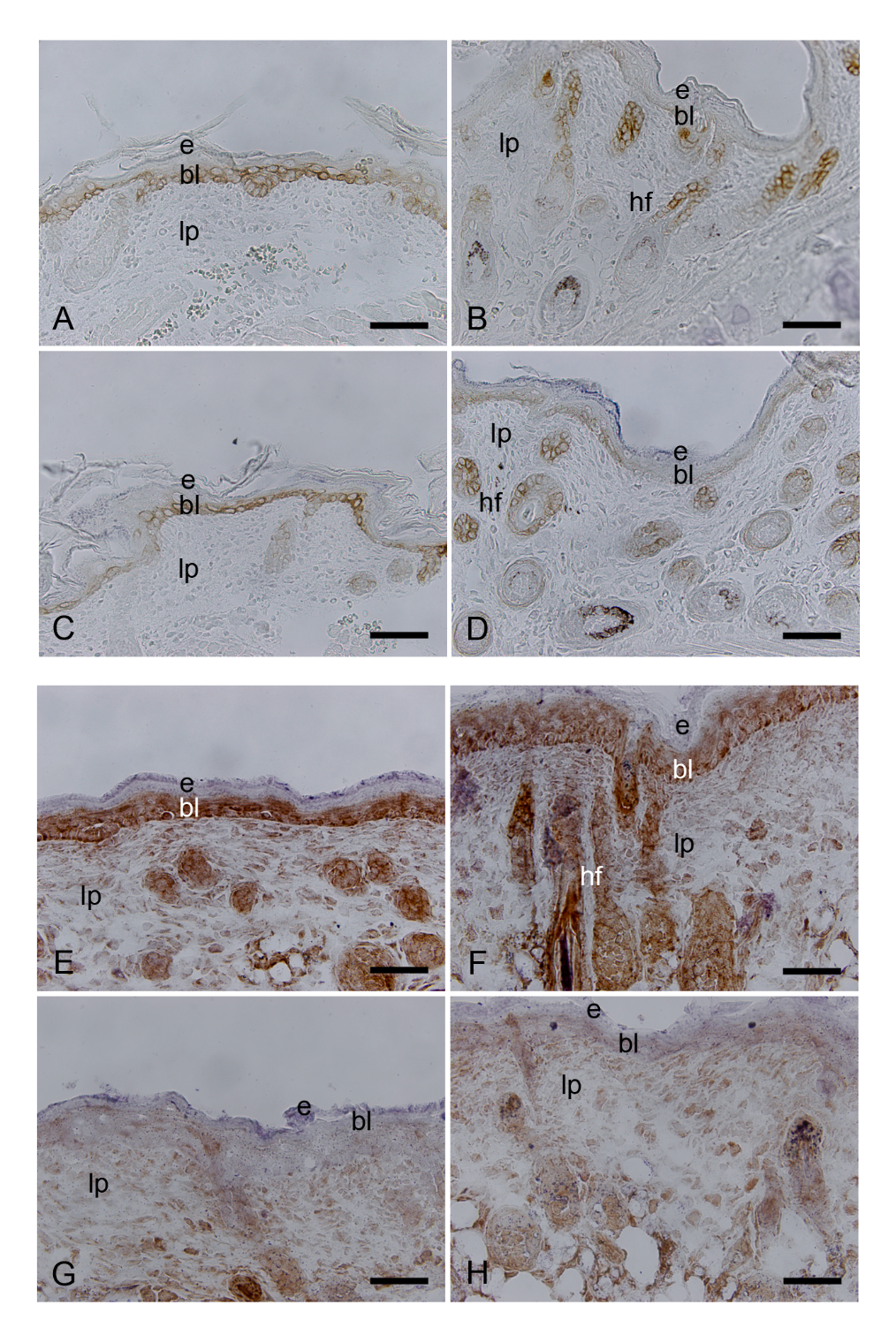


SFigure 3


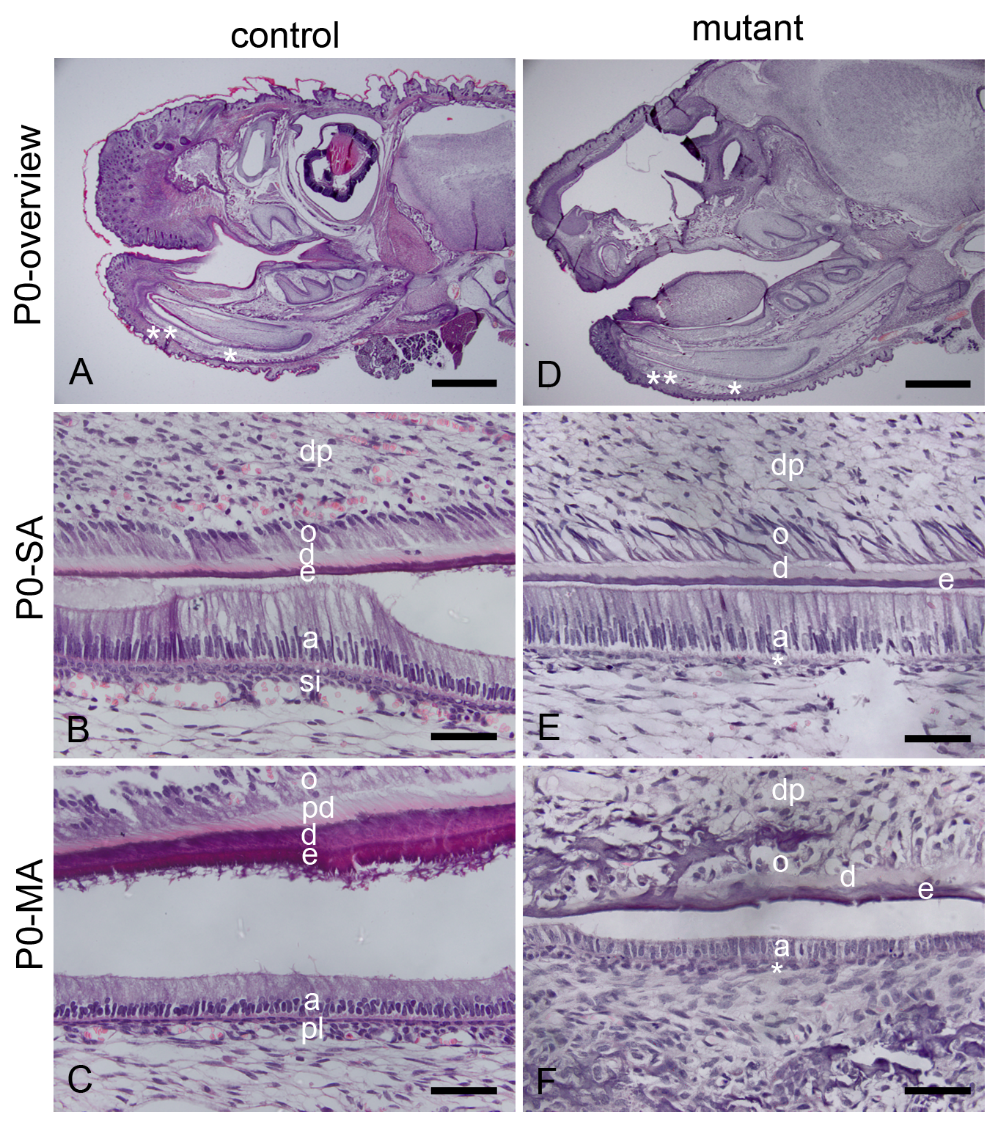


SFigure 4


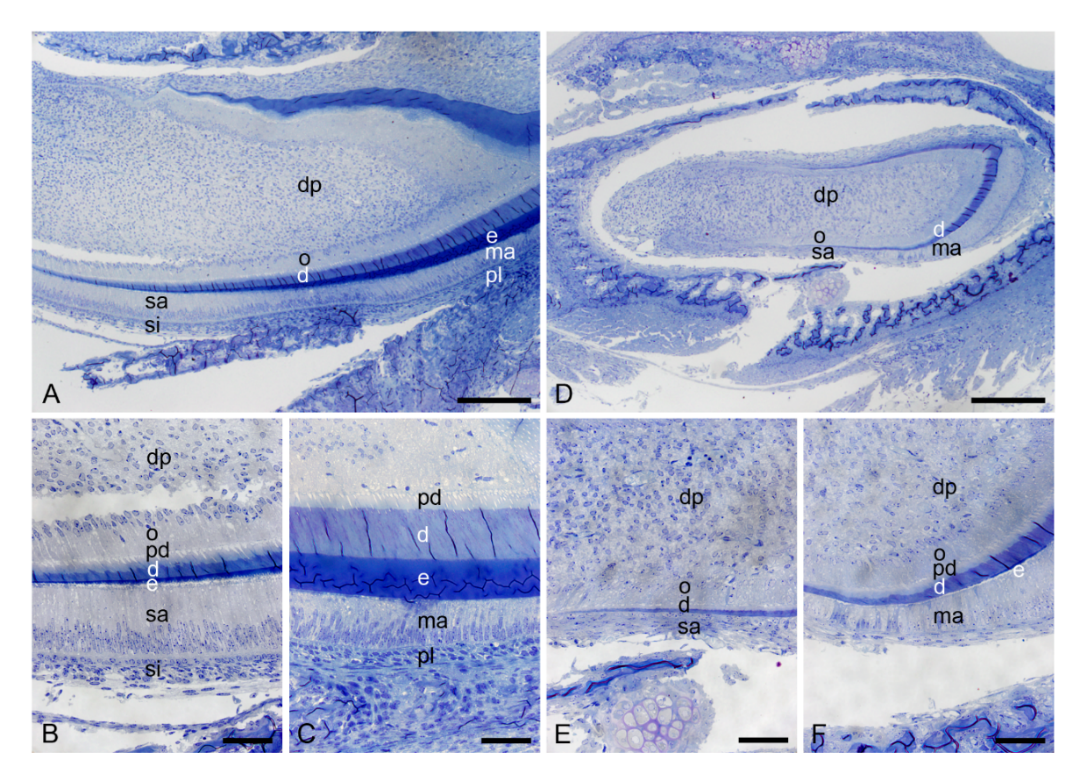


SFigure 5


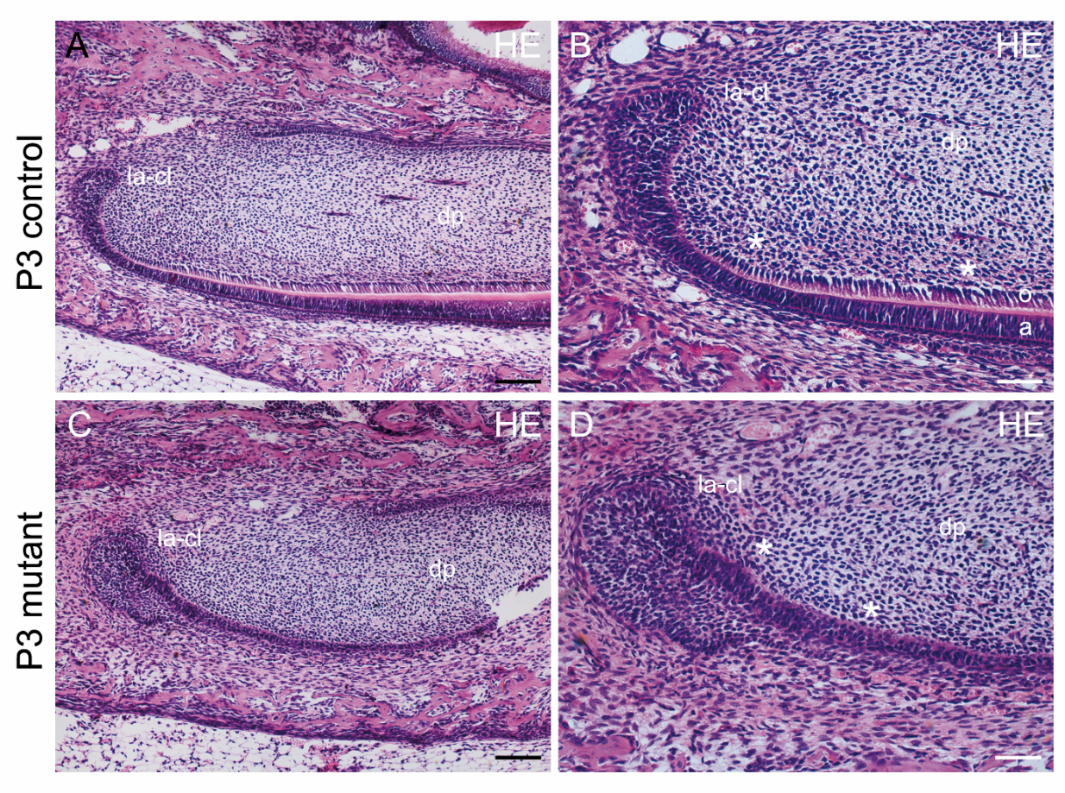

Supplement: Supplementary file 1 — Supplementary Figures. [file 41598_2020_77954_MOESM1_ESM.docx]
